# Supplementary material for: Social networks and COVID-19 vaccination intention in Dutch middle-aged and older adults in 2020: Insights into individual, interpersonal, community, and societal determinants – The SaNAE study
Source: Vaccine X. 2024 Sep 24;20:100562. doi: 10.1016/j.jvacx.2024.100562 (PMC11466667; doi:10.1016/j.jvacx.2024.100562)
Supplement: Supplementary Data 1 [file mmc1.docx]

**Supplementary Table 1.** Overview of social network characteristics described by structure, function, and quality

| Social network structure | |
| --- | --- |
| Network size and social isolation | Respondents could report up to fifteen family members, ten friends, ten acquaintances, five other network members, and five healthcare professionals (HCP). The sum of all network members per relationship type provided the total network size, resulting in a maximum of 45 persons. Network size was then further categorized based on quartiles: 0-4, 5-8, 9-13, and more than 13 network members. Network size was categorized to provide a quantitative measure for social isolation (0-4 network members) |
| Partner | Partner was assessed by a single question: do you have a partner? Answer categories included yes or no. |
| Type of relationships | The proportions of types of relationships were calculated by dividing the number of family members by the total network size. The same was performed for friends, acquaintances, other network members, and healthcare professionals. |
| Network diversity | Social network diversity was constructed based on the relationship types reported and included the following categories:   - Having no family, but friends, acquaintances, others, or HCP; - Having only family members; - Having only family members and friends; - Having family members, others, HCP, but no friends - Having family members, friends, acquaintances, other network members, and HCP. |
| Network density | Respondents could answer a 5-scaled statement about whether their friends and family know each other. |
| Homogeneity in gender and age | The proportion of network members of the same gender was calculated by dividing the number of network members of the same gender by the total network size. The proportion of network members of the same age (~5-year age range) was calculated by dividing the number of network members of the same age by the total network size. |
| Contact with children aged five years and younger | Contact with young children aged five years and younger was assessed by a single question with answer categories: yes daily, yes weekly, yes monthly, yes less often, no. Answer categories yes daily and yes weekly were combined into yes, daily, or weekly, and categories yes monthly and yes less often were combined into yes, monthly, or less often. |
| Living alone | The living situation was assessed by a single question with several answer categories: living alone, living with a partner, living with children, living with parents, living with other adults. Living alone was constructed based on the answer category: living alone. |
| Geographical proximity | Proportions of network members who live in the same house, within walking distance, less than 30 minutes away by car, more than 30 minutes away by car, or further away were calculated by dividing the number of network members living in the same house by the total network size, and so on. |
| Social network function | |
| Informational support | The proportion of informational supporters was calculated by dividing the number of network members who advised on problems or gave information by the total network size. |
| Emotional support | The proportion of emotional supporters was calculated by dividing the number of network members who provided the opportunity to discuss important matters or health-related topics by the total network size. |
| Practical support | The proportion of practical supporters was calculated by dividing the number of network members who helped with small or larger tasks in or around the house by the total network size. |
| Social network quality | |
| Social strain | The proportion of network members with whom social strain was experienced was calculated by dividing the sum of network members who are demanding, straining, or criticizing by the total network size. |
| Good relationships | The proportion of network members with whom there is a good relationship was calculated by dividing the number of social network members with whom there is a good relationship by the total network size. |
